# Nanopore 16S sequencing enhances the detection of bacterial meningitis after neurosurgery

**DOI:** 10.1002/acn3.51517

**Published:** 2022-02-06

**Authors:** Yoonhyuk Jang, Seondeuk Kim, Narae Kim, Hyoshin Son, Eun Jin Ha, Eun Jung Koh, Ji Hoon Phi, Chul‐Kee Park, Jeong Eun Kim, Seung‐Ki Kim, Sang Kun Lee, Won‐Sang Cho, Jangsup Moon, Kon Chu

**Affiliations:** ^1^ Department of Neurology Seoul National University College of Medicine Seoul National University Hospital Seoul South Korea; ^2^ Department of Neurosurgery Seoul National University College of Medicine, Seoul National University Hospital Seoul South Korea; ^3^ Division of Pediatric Neurosurgery Seoul National University Children's Hospital Seoul South Korea; ^4^ Department of Genomic Medicine Seoul National University Hospital Seoul South Korea

## Abstract

**Objective:**

Nosocomial bacterial meningitis is one of the major complications after neurosurgery. We performed nanopore 16S amplicon sequencing from cerebrospinal fluid (CSF) to evaluate bacterial meningitis in patients who underwent neurosurgery.

**Methods:**

Among the patients who visited the neurosurgery department of Seoul National University Hospital between July 2017 and June 2020, those with clinically suspected bacterial meningitis were included. 16S rDNA PCR was performed from the CSF, and nanopore sequencing was performed for up to 3 h. The reads were aligned to the BLAST database. In each case, the culture and the 16S rRNA gene amplicon analysis were simultaneously performed and compared with each other, and we retrospectively reviewed the medical records. Genuine infection was determined by the identical results between conventional culture study and the sequencing, or clinically determined in cases with inconsistent results between the two methods.

**Results:**

Of the 285 samples obtained from 178 patients who had 16S rDNA PCR, 41 samples (14.4%) were diagnosed with genuine infection. A total of 56.1% (23/41) of the samples with genuine infection showed a false‐negative culture test. In particular, 16S amplicon sequencing was useful in evaluating patients at the initial tests who had infection with intraventricular hemorrhage (Culture false‐negative rate = 100%), subarachnoid hemorrhage (Culture false‐negative rate = 77.8%), and systemic cancer (Culture false‐negative rate = 100%), which are risk factors for central fever. Moreover, 16S amplicon sequencing could suggest the possibility of persistent bacterial meningitis in empirical antibiotic use.

**Conclusion:**

CSF nanopore 16S sequencing was more effective than conventional CSF culture studies in postoperative bacterial meningitis and may contribute to evidence‐based decisions for antibiotic maintenance and discontinuation.

## Introduction

Nosocomial bacterial meningitis is one of the dangerous complications after neurosurgery.^1^, ^2^ Fever, an important sign of infection, is common in patients in neurocritical care units and, in addition to infection, is caused by a variety of factors, such as postoperative atelectasis, central origin, and drug side effects.^3^, ^4^, ^5^ Moreover, prophylactic antibiotics, generally administered to patients before neurosurgery,^6^, ^7^, ^8^ could make it particularly difficult to identify the cause of the fever because the antibiotics can affect culture yield or cause drug fever. Therefore, when postoperative fever appears, clinicians are challenged to determine maintenance or discontinuation of empirical antibiotics due to uncertainty of the cause. Notably, culture‐negative infections are often diagnosed in patients,^9^ but no objective criteria exist to confirm the infection.

Nanopore 16S amplicon sequencing is characterized by high sensitivity and specificity and an effective method to detect a pathogen in metagenomics research.^10^, ^11^, ^12^ In fact, 16S amplicon sequencing has demonstrated its capability in various clinical cases,^13^, ^14^, ^15^, ^16^, ^17^ and we also demonstrated its usefulness for bacterial meningitis in cerebrospinal fluid (CSF) by showing that 16S amplicon sequencing has advantages in the rapid detection of pathogens and the sensitivity after the prior use of antibiotics.^18^, ^19^ Thus, nanopore 16S amplicon sequencing has the potential to be practical in cases of suspected infection after neurosurgery as the hastening and sensitive detection of pathogens in central nervous system (CNS) would be helpful in the decision of antibiotics maintenance and stewardship.

Here, we aimed to study the utility of nanopore 16S amplicon sequencing when bacterial meningitis was suspected in patients who underwent neurosurgery. Moreover, we investigated risk factors associated with bacterial meningitis and the situations in which nanopore 16S amplicon sequencing is particularly useful.

## Methods

### Patient enrollment and sample collection

We included patients who were admitted to the neurosurgery department of Seoul National University Hospital from December 2017 to July 2020. Clinicians suspected a patient's bacterial meningitis in the situations, such as fever, CSF leakage, wound problems, neurologic changes, and change of CSF profile. Samples of CSF were collected and placed in sterile tubes for culture and sequencing. The bottles for the culture study were immediately transported to the microbiology laboratory. The sequencing test was performed only during working hours on weekdays; therefore, the bottles for sequencing were stored at 4°C before the experimental procedures. The conventional culture study and 16S PCR were performed simultaneously in each case.

### Medical records review

All the analyses in this study were conducted in a retrospective, case–control, and single‐center trial. The patients' medical records were retrospectively reviewed by three neurologists (Y.J., S.K., and J.M.) and included the neurologic diagnosis, the type of neurosurgery a patient underwent, risk factors, patients' symptoms or signs, and CSF profile. Foreign bodies applied to the CNS included a ventriculoperitoneal (VP) shunt, ommaya insertion, and intracranial pressure monitor, which could have been a source of bacterial meningitis. External ventricular drainage (EVD) was not included as a CNS foreign body because it was thought to have been applied after bacterial meningitis had occurred. Heavy alcohol consumption was defined according to the guidelines of the substance abuse and mental health services administration (SAMHSA).^20^ This study was approved by the Seoul National University Hospital Institutional Review Board (No. 1903‐060‐1016 and 1801‐135‐918). Written informed consent was provided by all patients and/or legal guardians.

### 16S amplicon analysis and determination of genuine infection

16S rDNA polymerase chain reaction (PCR) and 16S amplicon analysis were performed as described previously.^11^ Detailed methods are described in Data S1. Clinicians in the neurosurgery department requested 16S amplicon analysis when bacterial meningitis was suspected, and the results were used as one of the indicators of CNS infection. Genuine infection was determined when the pathogens identified by the conventional culture study and the sequencing were identical. In cases with inconsistent results between the two methods, genuine infection was determined prospectively by field clinicians (E.J.H., J.H.P., C‐K.P., J.E.K., S‐K.K., and W‐S.C.) and again, retrospectively reviewed by three neurologists (Y.J., S.K., and J.M.) according to the criteria, which consist of vital sign changes, the laboratory results of blood and CSF, and the patients' clinical course. If there was a discordant among the reviewers' opinions, the three reviewers discussed the cases together, and if required, the reviewers asked again the field clinicians (co‐authors, H.S., E.J.H., E. J. K., J.H.P., C‐K.P., J.E.K., S‐K. K, and W‐S. C.) their opinions to make a consensus.

### Statistical analysis

Clinical data were summarized as percentages or means ± standard deviation. In the analysis of clinical data, we utilized the chi‐square or Fisher's exact test for categorical variables and the Student's *t*‐test for continuous variables. Multivariable logistic regression analysis was performed with parameters showing a *p*‐value <0.10 on univariable analysis. In logistic multiple comparison, multiple test correction was performed through the multi‐step correction method, Bonferroni correction. All analyses were performed using R version 4.0.2 (R Foundation for Statistical Computing, Vienna, Austria). A *p*‐value <0.05 was considered statistically significant.

## Results

### The characteristics of the collected samples

**Figure 1:**
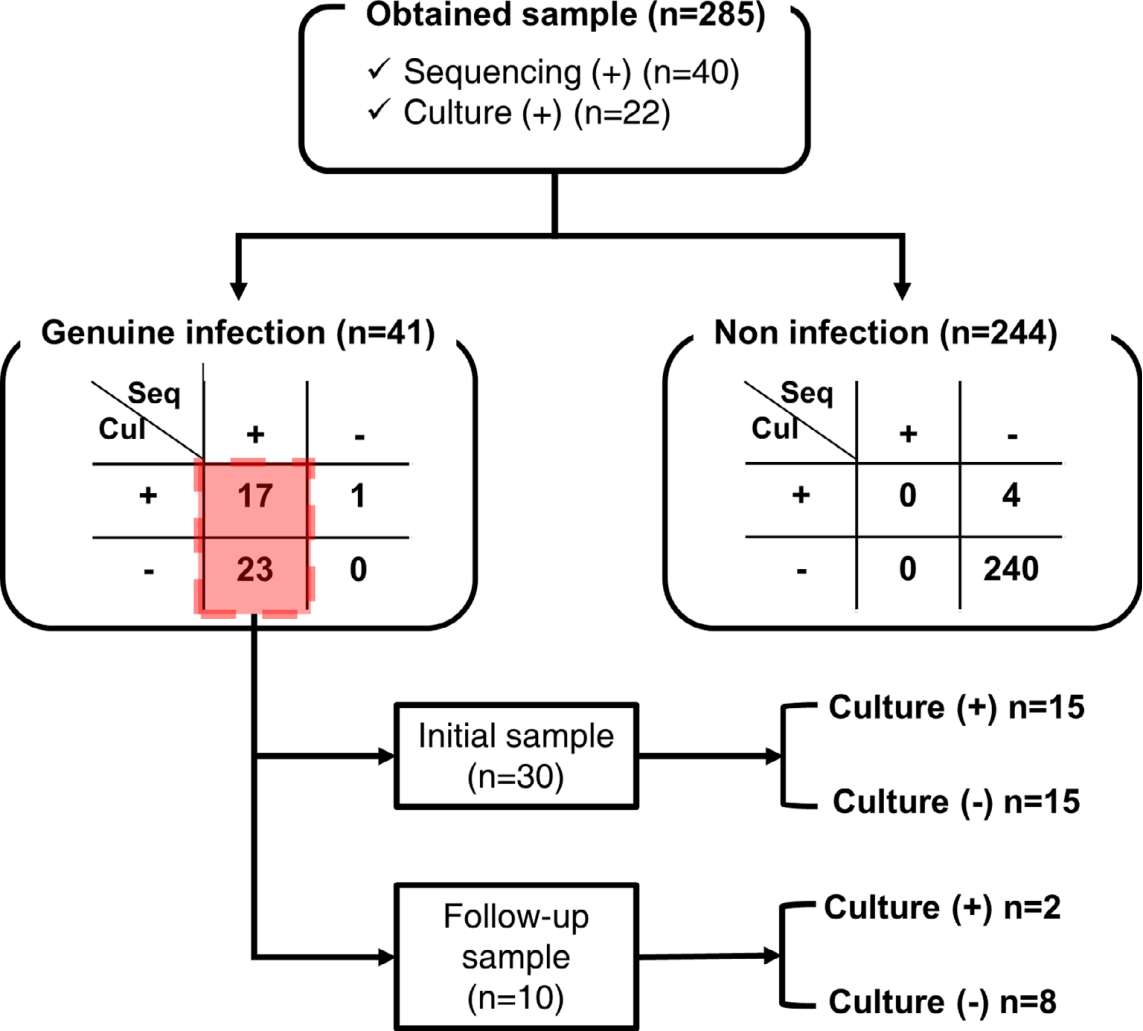
Study cohort profiles. Two hundred eighty‐five samples for nanopore 16S amplicon sequencing were obtained from 178 patients who underwent neurosurgery. Among them, 40 samples were sequencing positive, and 23 samples were culture positive. While all 40 sequencing‐positive samples were genuine infection, 18 culture‐positive samples were identified as genuine infection, and five were suspected of being contaminated. The final number of samples that were genuine infection was 41. Among the 40 sequencing‐positive samples, only 17 samples showed consistent results with the culture study, and 23 samples showed false‐negative results of the culture study. These were also divided into groups of initial samples (*n* = 30) and follow‐up samples (*n* = 10) depending on when the samples were collected. In each group, the numbers of false‐negative results of the culture study were 15 and 8, respectively. [Colour figure can be viewed at wileyonlinelibrary.com]

A total of 178 patients were tested via 16S rDNA PCR after neurosurgery (Fig. 1). The mean age of the patients was 52.7 years (range 1 to 88 years), and 99 patients (55.6%) were male. From the 178 patients, 285 samples were obtained, and among them, 40 samples and 22 samples were tested positive on 16S PCR and the conventional culture study, respectively. In the case of PCR positivity, we performed nanopore 16S amplicon sequencing. We thoroughly reviewed the medical records of each case, and we concluded that 41 samples (14.4%) were genuine infection (Fig. 1). All the positive test samples were monomicrobial infections. *Staphylococcus* was the most common species (*n* = 20, *S.aureus* = 5, *S.caprai* = 5, *S.epidermis* = 5, *S.hominis* = 3, *S.capitis* = 2), followed by *Klebsiella* (*n* = 6, *K.pneumoniae* = 3, *K.aerogenes* = 3). The detailed profile of the isolated bacteria is shown in the Table S1. Among the 41 cases, 40 samples (97.6%) were PCR positive, and only one sample (2.4%) had a false‐negative PCR result with microbial culture positivity of *Pseudomonas aeruginosa*. More than half (23/40, 56.1%) of the PCR‐positive samples showed a false‐negative microbial culture test. Meanwhile, four samples that showed positive culture tests and negative results on 16S sequencing were confirmed to be false‐positive results after re‐examinations. As the bacteria detected in the culture tests were common skin flora (*P.aeuroginosa, S.aureus, Unidentified Gram (+) rod, S.epidermidis*), we suspect that the samples were contaminated during the sample extraction or transport. Of the PCR‐positive samples, 75% (*n* = 30) of the samples were collected at the initial test when the patients had symptoms or signs, and 25% (*n* = 10) of the samples were collected at the follow‐up test. The false‐negativity ratio of the microbial culture was higher in the follow‐up samples (8/10, 80%) than in the initial samples (15/30, 50%).

### Risk factors associated with postoperative bacterial meningitis based on nanopore 16S amplicon sequencing

Next, we analyzed the risk factors associated with genuine postoperative infection, which was determined in combination with culture studies and 16S sequencing results. The mean age of the patients who were diagnosed with bacterial meningitis was 50.4 years (range 2 to 80 years), and 17 patients (63.0%) were males (Table 1). For the multivariable analysis, age, sex, the presence of CNS foreign bodies, heavy alcohol consumption, postoperative fever, and aggravation in the neurologic examination such as altered mentality and muscle weakness were included. Among them, the presence of CNS foreign bodies (*p* = 0.003, OR 3.311, 95% CI 1.509–7.246), heavy alcohol consumption (*p* = 0.022, OR 3.422, 95% CI 1.191–9.868), and postoperative fever (*p* = 0.004, OR 4.880, CI 1.815–16.091) were significantly associated with postoperative bacterial meningitis.

**Table 1:** Characteristics of samples from neurosurgery and association with risk factors.

Total	None (*n* = 244)	Genuine Infection (*n* = 41)	*p*‐value	Univariable analysis		Multivariable analysis	
				OR (95% CI)	*p* value	OR (95% CI)	*p* value
Patients	*n* = 153	*n* = 27					
Age (yrs)	49.9 ± 21.7	50.4 ± 23.8	0.756	1.002 (0.989–1.016)	0.741	1.002 (0.985–1.019)	0.819
Sex (Male)	81 (52.9%)	17 (63.0%)	0.230	1.60 (0.805–3.331)	0.191	0.845 (0.334–2.133)	0.720
Risk factors							
CNS Foreign Body	52 (21.3%)	17 (41.5%)	0.009*	2.615 (1.294–5.211)	0.00653*	3.311 (1.509–7.246)	0.003*
Alcoholics	25 (10.3%)	10 (24.4%)	0.018*	2.826 (1.197–6.316)	0.0135*	3.422 (1.191–9.868)	0.022*
Systemic cancer	15 (6.1%)	5 (12.2%)	0.182	2.120 (0.657–5.857)	0.169		
HTN	88 (36.1%)	16 (39.0%)	0.728	1.135 (0.566–2.222)	0.716		
DM	32 (13.1%)	6 (14.6%)	0.804	1.136 (0.405–2.749)	0.791		
Insulin	5 (2.1%)	0	1		NA		
Craniotomy	87 (35.6%)	11 (26.8%)	0.293	0.662 (0.3040–1.350)	0.273		
Diagnosis							
Brain tumor	80 (32.8%)	12 (29.3%)	0.721	0.848 (0.398–1.713)	0.656		
EDH	6 (2.5%)	1 (2.4%)	1	0.992 (0.052–6.018)	0.994		
SDH	25 (10.3%)	3 (7.3%)	0.778	0.692 (0.159–2.099)	0.562		
SAH	73 (29.9%)	12 (29.3%)	1	0.969 (0.454–1.963)	0.933		
IPH	57 (23.4%)	8 (19.5%)	0.690	0.795 (0.326–1.743)	0.587		
IVH	70 (28.7%)	8 (19.5%)	0.260	0.603 (0.249–1.310)	0.226		
Vascular occlusion	4 (1.6%)	1 (2.4%)	0.543	1.500 (0.076–10.463)	0.72		
Aneurysm	4 (1.6%)	0	1		NA		
Congenital defect	6 (2.5%)	2 (4.9%)	0.323	2.034 (0.291–9.198)	0.395		
Hydrocephalus	17 (7.0%)	6 (14.6%)	0.117	2.289 (0.783–5.936)	0.103		
Shunt malfunction	41 (16.8%)	15 (36.6%)	0.005*	2.856 (1.370–5.818)	0.0042*		
Hemifacial spasm	3 (1.2%)	0	1		NA		
Other diagnosis	2 (0.8%)	0	1		NA		
Symptom or sign							
postOP fever	175 (71.7%)	36 (87.8%)	0.033*	2.839 (1.163–8.524)	0.0361*	4.880 (1.815–16.091)	0.004*
CSF leakage	14 (5.7%)	4 (9.8%)	0.305	1.776 (0.483–5.269)	0.334		
Wound problem	25 (10.3%)	2 (4.9%)	0.392	0.449 (0.071–1.593)	0.289		
Neurologic change	21 (8.6%)	8 (19.5%)	0.047*	2.574 (1.003–6.106)	0.0379*	2.618 (0.904–7.241)	0.067
Other reasons	24 (9.8%)	3 (7.3%)	0.778	0.724 (0.166–2.204)	0.612		

CI, confidence interval, yrs., years, CNS, central nervous system, HTN, hypertension, DM, diabetes mellitus, EDH, epidural hemorrhage, SDH, subdural hemorrhage, SAH, subarachnoid hemorrhage, IPH, intraparenchymal hemorrhage, IVH, intraventricular hemorrhage, postOP, postoperative

* *p* < 0.05.

### Situations in which nanopore 16S amplicon sequencing was particularly useful

To evaluate the situations in which nanopore 16S amplicon sequencing was particularly useful, we analyzed the 30 samples obtained from 30 patients who were diagnosed with genuine infection at the initial test by nanopore 16S amplicon sequencing (Table 2). Of the 30 samples, 15 cases (15/30, 50%) had false‐negative microbial culture results. Most of the cases were accompanied by postoperative fever (12/15, 80%), and 16S amplicon sequencing was the only diagnostic tool that detected the pathogens in almost half of the cases with postoperative fever (12/25, 48%).

**Table 2:** Analysis of initial samples with nanopore 16S sequencing positive and the culture test false‐negative rate.

Total	Culture negative (*n* = 15)	Culture positive (*n* = 15)	Culture test false‐negative rate	*p*‐value
Patients	*n* = 15	*n* = 15		
Age (yrs)	47.5 (sd 7.4)	49.6 (sd 6.4)		0.833
Sex (Male)	10 (66.7%)	10 (66.7%)		1
Risk factors				
CNS Foreign Body	3 (20%)	9 (60%)	25%	0.06
Alcoholics	4 (26.7%)	4 (26.7%)	50%	1
Systemic cancer	3 (20%)	0	100%	0.224
HTN	7 (46.7%)	5 (33. 3%)	58.3%	0.71
DM	3 (20%)	2 (13.3%)	60%	1
Insulin	0	0	NA	NA
Craniotomy	4 (26.7%)	5 (33.3%)	44.4%	1
Diagnosis				
Brain tumor	5 (33.3%)	4 (26.7%)	55.6%	1
EDH	0	0		NA
SDH	1 (6.7%)	1 (6.7%)	50%	1
IPH	4 (26.7%%)	2 (13.3%)	66.7%	0.651
SAH	7 (46.7%)	2 (13.3%)	77.8%	0.109
IVH	7 (46.7%)	0	100%	0.006*
Vascular occlusion	0	1 (6.7%)	0	1
Aneurysm	0	0	NA	NA
Congenital defect	0	1 (6.7%)	0	1
Hydrocephalus	3 (20%)	2 (13.3%)	60%	1
Shunt malfunction	3 (20%)	8 (53.3%)	27.3%	0.128
Hemifacial spasm	0	0	NA	NA
Other diagnosis	0	0	NA	NA
Symptom or sign				
postOP fever	12 (80%)	13 (86.7%)	48%	1
CSF leakage	1 (6.7%)	3 (20%)	25%	0.598
Wound problem	0	2 (13.3%)	0	0.483
Neurologic change	4 (26.7%)	1 (6.7%)	80%	0.33
Other reasons	1 (6.7%)	1 (6.7%)	50%	1

yrs, years, sd, standard deviation, CNS, central nervous system, HTN, hypertension, DM, diabetes mellitus, EDH, epidural hemorrhage, SDH, subdural hemorrhage, IPH, intraparenchymal hemorrhage, SAH, subarachnoid hemorrhage, IVH, intraventricular hemorrhage, postOP, postoperative.

* *p* < 0.05.

In the cases of intraventricular hemorrhage (IVH) (7/15 [46.7%] vs. 0, *p* = 0.006) and systemic cancer (3/15 [20%] vs. 0, *p* = 0.224), all bacterial meningitis was detected only by 16S amplicon sequencing. While statistical significance was not achieved, the probability of obtaining false‐negative results on culture studies was more than 70% in the diagnosis of subarachnoid hemorrhage (SAH) and the patients' neurologic aggravation.

Meanwhile, the cases with CNS foreign bodies, shunt malfunction, and CSF leakage showed less than 30% of probability of obtaining inconsistent results between culture studies and 16S amplicon sequencing.

### Analysis of individual patients who showed false‐negative culture results

We further investigated each case of the 17 patients who showed false‐negative culture results in which nanopore 16S amplicon sequencing confirmed bacterial meningitis (Table 3). The patients were categorized into three groups: (A) patients diagnosed with CNS infection accompanied by systemic infection, (B) patients diagnosed with isolated CNS infection without systemic infection, and (C) patients who showed false‐negative results on culture studies at follow‐up samples. The 16S amplicon sequencing results helped in clinical decisions such as antibiotic changes, antibiotic maintenance, wound revision, or foreign body removal. We described representative cases from each group below.

**Table 3:** Detailed profile of patients who had mismatch results between microbial culture and nanopore 16S sequencing.

Patient ID	Sex/age	Diagnosis	Operation	Risk factors	Reason for nanopore sequencing	Day (OP to CSF study)	CSF profiles	Systemic infection	Blood or other system culture result	CSF culture result	16S sequencing Result	ABX	Change of actions, based on 16S Seq
Group A: CNS infection accompanied by Systemic infection, confirmed by nanopore 16S sequencing													
A#1	M/75	Ventriculitis with IVH	EVD	Pancreatic head cancer	Progression of Hydrocephalus with neurologic change	0	R81 W900(P90 L7 O3), Ptn665 Glc23 Glc ratio 0.15	Sepsis, UTI, Liver abscess	*Klebsiella pneumoniae* (Ceftriaxone S): Blood/Urine/PCD	Negative	*Klebsiella pneumoniae*	Vancomycin, Ceftazidime, Metronidazole ➔ Ceftriaxone	ABX narrow down
A#2	F/80	SAH, ICH	Coil embolization of cerebral aneurysm	Alcoholics, HTN, DM, Lumbar catheter	Postop fever	7	R1725 W1170(P91 L6 O3), Ptn212, Glc49 Glc ratio0.26	Sepsis, UTI	*Klebsiella pneumoniae* ESBL (+) (Meropenem I): Blood/Urine/Tip	Negative	*Klebsiella pneumoniae*	Meropenem, Gentamycin ➔ IV/IT Colistin	ABX escalation
A#3	M/51	SAH, IVH	EVD	Alcoholics, HTN, DM	Postop fever	20	R1 W3960(P90 L1 O9), Ptn343, Glc64 Glc ratio 0.32	Sepsis (CRBSI)	*Enterobacter cloacae* (Cefepime S): Blood/Tip	Negative	*Enterococcus faecium*	Vancomycin, Cefepime ➔ Linezolid	ABX escalation
A#4	M/56	Metastatic cancer to the brain	Craniotomy and tumor removal	Left frontal sinus cancer	Postop fever	9	R12 W11(P18 L64 O18) Ptn74 Glc 74 Glc ratio NA	Pneumonia	*Klebsiella pneumoniae* (Ceftriaxone S): Sputum only	Negative	*Klebsiella pneumoniae*	Vancomycin, Ceftazidime ➔ Metronidazole, Ceftriaxone	ABX change
A#5	M/63	ICH, IVH	EVD	Alcoholics, Rectal cancer	Postop fever	14	R870 W580(P53 L12 O35), Ptn82 Glc110 Glc ratio NA	Cellulitis	*Staphylococcus hominis* (Oxacillin R): Skin only	Negative	*Staphylococcus hominis*	Vancomycin, Ceftazidime	ABX maintenance
A#6	M/60	Tuberculum sellae meningioma	Endoscopic extended skull base TSA and tumor removal	Alcoholics, HTN	Postop CSF leak	12	R NA W18600(P87 L0 O13) Ptn574 Glc1 Glc ratio < 0.01	Sepsis	*Streptococcus pneumoniae* (Ceftriaxone R, Vancomycin S): Blood	Negative	*Streptococcus pneumoniae*	Vancomycin, Ceftazidime	Abx maintenance
Group B: isolated CNS infection, confirmed by nanopore 16S sequencing													
B#1	M/7	Cerebellar pilocytic astrocytoma	Craniotomy and tumor removal	None	Postop fever	12	R403 W5584(P81 L4 O15) Ptn143 Glc59 Glc ratio NA	None	Negative	Negative	*Staphylococcus epidermidis*	Vancomycin, Ceftazidime ➔ Vancomycin	ABX narrow down
B#2	M/1	Germ cell tumor, immature teratoma, intracranial	Shunt revision	VP shunt	Postop fever	3	R432 W5490(P88 L2 O10) Ptn600 Glc < 5 Glc ratio NA	None	Negative	Negative	*Klebsiella aerogenes*	Vancomycin, Ceftazidime ➔ Vancomycin, Cefepime	ABX escalation, VPS removal
B#3	F/71	SAH	Craniotomy and cerebral aneurysm clipping	HTN	Postop fever	5	R80000 W900(P91 L5 O4), Ptn268 Glc40 Glc ratio 0.39	None	Negative	Negative	*Staphylococcus caprae*	Vancomycin, Ceftazidime	ABX maintenance, Wound revision & bone flap removal
B#4	M/15	Diffuse midline glioma	VP shunt	VP shunt	Neurologic change (mental change)	23	R390 W20(P10 L20 O70) Ptn148 Glc91 Glc ratio NA	None	*Staphylococcus epidermidis* (contamination): Tip	Negative	*Staphylococcus aureus*	Vancomycin, Meropenem	ABX maintenance
B#5	F/32	SAH, IVH	EVD	None	Postop fever	4	R92000 W1386(P89 L8 O3), Ptn500 Glc2 Glc ratio NA	None	Negative	Negative	*Enterococcus faecium*	Vancomycin, Ceftazidime	ABX maintenance
B#6	F/69	SAH, IVH	EVD	HTN, DM	CSF abnormality	3	R174000 W2598(P77 L5 O18) Ptn 132 Glc 103 Glc ratio 33.0	None	Negative	Negative	*Moraxella osloensis*	None	None
Group C: follow‐up sample mismatch result, confirmed by nanopore 16S sequencing													
C#1	F/63	Metastatic cancer to the brain	Ommaya insertion	Breast cancer, Ommaya	Postop fever	7	R77 W135(P29 L22 O49) Ptn 38 Glc 59 Glc ratio NA	None	Negative	Unidentified Gram (+) rod (Vancomycin S) ➔ Negative (FU)	*Corynebacterium pilbarense (FU)*	Vancomycin, Ceftazidime ➔ Vancomycin	ABX narrow down & maintenance
C#2	M/38	Ventriculitis	EVD	None	Postop fever Altered mentality	3	R477 W234(P68 L28 O4) Ptn 334 Glu 22 Glc ratio NA	None	Negative	*Pseudomonas aeruginosa* (Ceftazidime S) ➔ Negative (FU)	*Pseudomonas aeruginosa (FU)*	Vancomycin, Ceftazidime ➔ Ceftazidime	ABX narrow down & maintenance
C#3	M/3	Hydrocephalus	VP shunt	VP shunt	Fever	1210	R54 W4(P100 L0 O0) Ptn24 Glc58 Glc ratio NA	None	Negative	*Staphylococcus aureus* (Oxacillin R) ➔ Negative (FU)	*Staphylococcus aureus* (FU)	Vancomycin, Rifampin	ABX maintenance, VPS removal
C#4	M/6	Holoprosencephaly	VP shunt	VP shunt	Fever	167	R100 W10(P20 L4‐ O40) Ptn 124 Glc 43 Glc ratio NA	None	Negative	*Staphylococcus epidermidis* (Oxacillin R) ➔ Negative (FU)	*Staphylococcus epidermidis* (Initial & FU)	Vancomycin, cefotaxime ➔ Vancomycin, Rifampin	ABX maintenance
C#5	F/79	SAH, ICH, IVH	Cranioplasty with autologous bone	HTN	Fever	8	R27700 W160(P18 L51 O31) Ptn 116 Glc 128 Glc ratio NA	None	Negative	*Staphylococcus capitis* (Oxacillin R) ➔ Negative (FU)	*Staphylococcus capitis* (Initial & FU)	Vancomycin, Meropenem ➔ Vancomycin	ABX maintenance

ID, identification, OP, operation, CSF, cerebrospinal fluid, ABX, antibiotics, Seq, sequence, CNS, central nervous system, M, male, F, female, IVH, intraventricular hemorrhage, EVD, extraventricular drainage, R, red blood cell, W, white blood cell, P, polymorphonuclear leukocytes, L, lymphocyte, O, others, Ptn, protein, Glc, glucose, Glc ratio, CSF glucose/serum glucose ratio, UTI, urinary tract infection, S, susceptible, I, intermediate, R, resistant, PCD, percutaneous drainage, SAH, subarachnoid hemorrhage, ICH, intracerebral hemorrhage, HTN, hypertension, DM, diabetes mellitus, Postop, postoperative, ESBL, extended‐spectrum β‐lactamase, IV, intravenous, IT, intrathecal, CRBSI, central‐related bloodstream infection, TSA, transsphenoidal approach, VP, ventriculoperitoneal, VPS, ventriculoperitoneal shunt, FU, follow‐up.

### Group A: CNS infection accompanied by systemic infection, confirmed by nanopore 16S amplicon sequencing

All six patients in Group A had negative microbial culture tests in the CSF samples, but nanopore 16S amplicon sequencing revealed the causative bacteria, consistent with culture test results in systemic samples such as blood, urine, sputum, skin, or catheters. Due to nanopore 16S amplicon sequencing, CNS involvement by systemic infection or vice versa was confirmed, and the use of antibiotics could be modulated according to the susceptibility test results of the systemic culture tests.

### Representative case in group A: *Klebsiella pneumoniae* meningoencephalitis (patient A#1)

**Figure 2:**
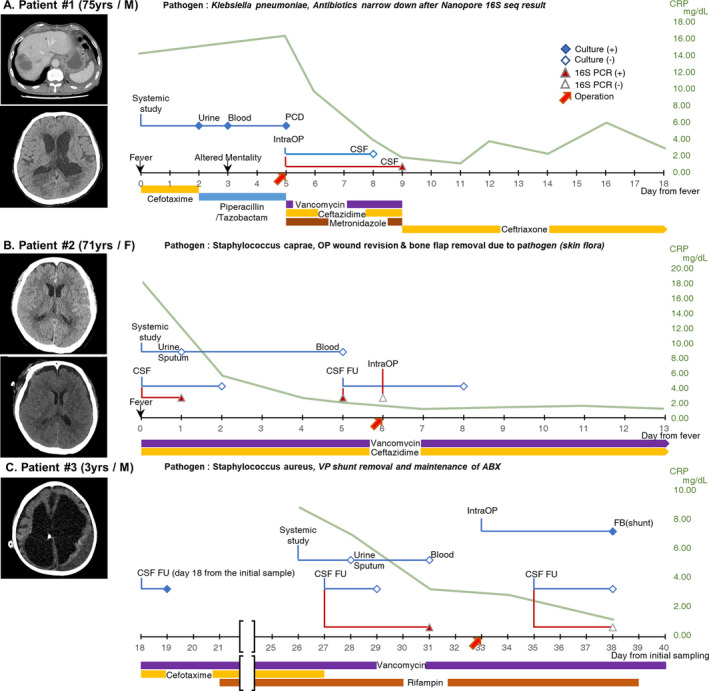
Representative Cases of 16S Sequencing Culture Mismatching. (A) A representative case of Group A. A 75‐year‐old man developed fever, and a systemic study revealed liver abscess (A‐1), complicated urinary tract infection, and sepsis. He showed altered mentality, and brain computed tomography (CT) revealed signs of ventriculitis (day 3) (A‐2). Thus, extraventricular drainage (EVD) was inserted (day 5). While the systemic culture study isolated 2nd generation cephalosporin susceptible *Klebsiella pneumoniae*, a negative result was shown in the culture study of cerebrospinal fluid (CSF) obtained from EVD. However, nanopore 16S amplicon sequencing detected *K. pneumoniae* (red‐filled triangle), which was a consistent result with that of the systemic culture study (day 9). Based on the susceptibility results of the systemic culture study, the empirical antibiotics were narrowed down to ceftriaxone monotherapy. (B) A representative case of Group B. A 71‐year‐old female had severe headache and was diagnosed with subarachnoid hemorrhage (SAH) due to aneurysmal rupture (B‐1). She underwent craniotomy and cerebral aneurysm clipping surgery. Postoperative fever occurred after 5 days (day 0). All conventional culture studies showed negative results, but only nanopore 16S amplicon sequencing of CSF detected *Staphylococcus caprae* (day 1). Brain CT supported the possibility of surgical site infection (B‐2). Therefore, wound revision and bone flap removal surgery were performed (day 6). (C) A representative case of Group C. A 3‐year‐old boy with a history of congenital hydrocephalus was diagnosed with ventriculoperitoneal (VP) shunt infection. An initial (day 0, not shown) and the first follow‐up (day 18) culture study of CSF samples isolated *Staphylococcus aureus*. While the additional follow‐up culture study showed a negative result, nanopore 16S amplicon sequencing detected *S. aureus* (day 27). The clinicians decided to remove the VP shunt (day 33), and the culture study with the removed shunt revealed *S. aureus,* the consistent pathogen with 16S amplicon sequencing. Therefore, antibiotic treatment was maintained until the final follow‐up study result (day 35), which was negative. PCD, percutaneous catheter drainage; IntraOP, intraoperation; CSF, cerebrospinal fluid; FU, follow‐up; FB, foreign body. [Colour figure can be viewed at wileyonlinelibrary.com]

A 75‐year‐old male with a history of pancreatic cancer and chemotherapy visited our emergency room because of a fever (Fig. 2A). It was determined that the fever was related to a liver abscess. Empirical ceftriaxone was administered, and the systemic culture results reported *Klebsiella pneumoniae* susceptibility to ceftriaxone. However, the fever was refractory, and the clinicians escalated the antibiotics to piperacillin and tazobactam. On day three after fever onset, the patient's mental status worsened with a Glasgow Coma Scale (GCS) score of E3VtM2. Brain CT revealed signs of ventriculitis. Therefore, extracranial ventricular drainage (EVD) was implemented on day five, and the clinicians empirically administered vancomycin, cefotaxime, and metronidazole. While the conventional culture of the intraoperative CSF sample could not prove the pathogen of CNS infection, 16S amplicon sequencing reported that *K. pneumoniae* matched the pathogen of the systemic samples. Therefore, the antibiotics were adjusted to ceftriaxone according to the susceptibility result of the systemic cultures. After antibiotics for 8 weeks, the patient's CRP level normalized, and his mental status fully returned to normal (GCS E4VtM6).

### Group B: isolated CNS infection, only confirmed by nanopore 16S amplicon sequencing

All six patients in group B showed isolated CNS infection. Neither CSF nor blood samples showed positive culture results, but 16S amplicon sequencing in CSF confirmed bacterial meningitis. In this group, the detection of pathogens that would not have been possible with conventional culture tests alone was achieved through nanopore 16S amplicon sequencing, and it was especially useful for the discrimination of genuine infection from central fever.

### Representative case in group B: *Staphylococcus caprae* meningitis (patient B#3)

A 71‐year‐old female was transferred to our hospital with severe headache (Fig. 2B). She had aneurysm clipping surgery due to SAH. Before the surgery, prophylactic antibiotic, cefazolin, administered until postoperative day four. A fever occurred on postoperative day five, and the CSF profile revealed a high density of cells with elevated protein, showing an RBC count of 80,000 cells/m^2^, WBC count of 2890 cells/m^2^ [91% neutrophil (N), 5% lymphocyte (L), 4% others (O)], protein of 268 (15–445 mg/m^2^), and CSF/serum glucose ratio of 38.5%. Thus, under suspicion of CNS infection, the clinicians empirically administered vancomycin and ceftazidime 4 h after the CSF sample extraction. Whereas the conventional culture studies were all negative in systemic and CSF samples which were obtained 48 h after the administration of prophylactic antibiotics, 16S amplicon sequencing was positive in the CSF, showing *Staphylococcus caprae*. On day five after fever onset, a follow‐up CSF 16S sequencing was consistent with the previous result. Follow‐up brain CT revealed soft tissue swelling and fluid collection at the operative site. Therefore, the operative site was suspected as the origin of infection, and the patient underwent wound revision surgery on day six from fever onset. With the maintenance of antibiotics, the patient gradually recovered with no focal neurologic deficit.

### Group C: follow‐up sample culture false‐negative results confirmed by nanopore 16S amplicon sequencing

Group C included five patients. The conventional culture test was positive in the initial samples; however, after antibiotic use, although the patients' clinical conditions did not fully recover, the results were negative in the follow‐up samples, which still showed positive results in 16S amplicon sequencing. Therefore, even under a low bacterial load, nanopore 16S amplicon sequencing was able to more sensitively detect pathogens that might not have been fully resolved.

### Representative case in group C: *Staphylococcus aureus* meningoencephalitis (patient C#3)

A 3‐year‐old boy was transferred to our intensive care unit because of progressively decreased responsiveness over 2 weeks. He had congenital hydrocephalus and a history of VP shunt. A CSF test was within normal limits, but under suspicion of VP shunt infection, empirical antibiotics were administered. In the initial (day 0) and follow‐up (day 18) samples, *Staphylococcus aureus* was grown with an aggravating course of the CSF profile showing mild pleocytosis (WBC 15 cells/m^2^, protein 12 mg/m^2^, and CSF/serum glucose ratio of 70%). After adding antibiotics, while the additional follow‐up culture study (day 27) was negative and the CSF profile revealed a normal WBC count (WBC 4 cells/m2 and protein 124 mg/m2), the patient's fever and altered mentality were not recovered. However, 16S amplicon sequencing revealed *S. aureus,* which was consistent with previous culture results. Therefore, the clinicians could not rule out bacterial meningitis and decided to remove the VP shunt, which was a possible origin of the infection. In fact, *S. aureus* was detected in the culture study with a VP shunt tip*,* confirming the result of 16S sequencing. Antibiotics were maintained until the final follow‐up study result (day 35), which was negative, and the patient's consciousness gradually recovered.

## Discussion

In our analysis with CSF nanopore 16S amplicon sequencing and the conventional CSF culture study, the cases after neurosurgery showed 14.4% genuine bacterial meningitis when the infection was suspected. Nanopore 16S amplicon sequencing was highly sensitive, detecting more than half of genuine infection cases alone. In particular, nanopore 16S amplicon sequencing was useful for evaluating the presence of pathogens in ambiguous situations, such as postoperative fever or follow‐up samples after antibiotic treatment. Therefore, 16S amplicon sequencing was instrumental in clinical decisions for postoperative care.

Nanopore 16S rRNA amplicon sequencing is a promising technology in the metagenomic sequencing field, alternating conventional culture study. 16S rRNA gene exists in every bacteria and can be used to study bacterial phylogeny and taxonomy. Therefore, by 16S rRNA gene amplicon sequencing, bacterial identification is available without culture.^14^ Compared to the shotgun metagenomics sequencing, which is randomly sequencing the whole microbial genome, 16S rRNA amplicon sequencing has demonstrated its higher sensitivity and lower error rate for bacterial identification.^21^ Previously, Illumina or Ion Torrent technology has been applied for 16S rRNA gene amplicon sequencing.^22^, ^23^ In contrast, we used nanopore sequencing in our study. Nanopore has several advantages over previous methods. First, the whole 16S rRNA (V1‐9 region) can be analyzed through long‐read sequencing, compared to previous technology such as Illumina or Ion Torrent, which could sequence only the partial region (V3‐4) of the 16S rRNA gene.^24^, ^25^, ^26^ Moreover, the library preparation is simple and fast and real‐time analysis is available, which can be helpful in making rapid clinical decisions.^27^

Our research could be a reference for the clinical validity of nanopore 16S amplicon sequencing with postoperative CSF samples. In our previous study,^19^ we confirmed 16S amplicon sequencing was comparable to culture study of bacterial meningitis CSF sample. Specifically, the pathogen detection time was shortened from several days (culture) to a half‐day (16S amplicon sequencing), and a small amount (200 μg) of the sample did not attenuate the sensitivity of 16S amplicon sequencing. Moreover, the prospective application was successful in two patients.^19^ Based on these previous results, we could conduct the current research, which supports the utility of 16S amplicon sequencing in real‐time clinical decisions from larger cases.

Nanopore 16S amplicon sequencing showed higher sensitivity than conventional culture studies in the detection of pathogens after neurosurgery. A total of 56.1% (23/41) of our results were confirmed by 16S sequencing alone, and only one case was detected by culture study alone. Once again, this result confirmed the superiority of 16S amplicon sequencing over the conventional culture test, even in CSF samples.^18^, ^19^, ^28^ Therefore, there is a possibility that the postoperative CNS infection rate might actually be higher than the results of previous studies, which showed 0.3%−8.9% based on culture studies.^29^, ^30^, ^31^ In addition, a CNS foreign body, heavy alcohol consumption, and postoperative fever that we analyzed as risk factors for postoperative bacterial meningitis were partly consistent with the known factors for nosocomial bacterial meningitis.^29^, ^32^ We expect the result to more accurately reflect the actual clinical situation because the detection of pathogens by 16S amplicon sequencing is less affected by the use of prophylactic antibiotics.^13^, ^16^, ^17^, ^19^

Due to its high sensitivity, nanopore 16S amplicon sequencing could help clinicians in ambiguous cases where the bacterial load is lowered. Prophylactic antibiotics are generally administered to patients before and during neurosurgery.^6^, ^7^, ^33^ Furthermore, patients in neurocritical care units are vulnerable to systemic infections such as pneumonia, urinary tract infection, and catheter‐related bloodstream infection, thus empirical antibiotics are frequently administered before the infection is confirmed.^34^ In this situation, nanopore 16S amplicon sequencing could more accurately detect pathogens at a far lower titer than the culture study. As analyzed, the presence of CNS foreign bodies and CSF leakage that generally increase the bacterial load resulted in a higher culture‐positive rate, whereas follow‐up samples after empirical antibiotic use tended to have more frequent culture‐negative infections. In Group A, we showed the usefulness of 16S amplicon sequencing for detecting pathogens when conventional culture studies had false‐negative results after antibiotic use for systemic infection.

In particular, 16S amplicon sequencing was useful for detecting pathogens in postoperative fever cases with abnormal CSF profiles. Fever occurs in more than 70% of patients in the neurocritical intensive care unit,^35^, ^36^ and to manage the patients, it is important to differentiate the infection from central fever.^34^ A CSF analysis could be a reference to suspect bacterial meningitis, but an abnormal CSF profile is common for postoperative cases.^37^, ^38^ Therefore, clinicians cannot help but depend on the conventional CSF culture test alone for infection diagnosis, although it has a low diagnostic yield due to empirical antibiotic use.^39^ Meanwhile, as our results showed, 16S amplicon sequencing further detected 48% (12/25) of the genuine infections in the cases with postoperative fever at the initial tests. Especially in cases with some risk factors for central fever (SAH, IVH, or systemic cancer),^3^, ^5^, ^40^ 16S amplicon sequencing was useful in diagnosing the infection, showing a false‐negative rate of more than 70% of the culture. Indeed, in Group B, we presented examples of how 16S amplicon sequencing could identify bacterial meningitis in patients, which could have been confused with central fever. Therefore, nanopore 16S amplicon sequencing could be an effective tool to evaluate postoperative fever.

Taken together, nanopore 16S amplicon sequencing could be one of the references for antibiotic maintenance and discontinuation in patients after neurosurgery. Determining the duration of use of antibiotics is one of the most difficult and important challenges in the clinical setting of postoperative care.^31^ In this situation, clinicians' decisions are the most important, and nanopore 16S amplicon sequencing can help their empirical judgment whether to continue or discontinue antibiotics with high sensitivity for bacterial detection. In Group C, despite the negative results from repeated culture studies, clinicians were uncertain regarding discontinuation of antibiotics because the patients' conditions had not sufficiently recovered. In these situations, the positive results of 16S amplicon sequencing provided information about the possibility of unresolved infections, and furthermore, the turnover of the sequencing results from positive to negative could suggest evidence of improved status.

Our study for nanopore 16S amplicon sequencing has several limitations. 16S amplicon sequencing itself does not yet determine complete resolution of bacterial infection because it can also detect DNA from dead bacteria.^41^ In addition, the negative PCR determination might not have been accurate because it is dependent on the inspector's visual view of the electrophoresis band. This study was conducted in a single tertiary institution, Seoul National University Hospital, so bias could have been present in the selection of patients and the identified pathogens. Therefore, 16S amplicon sequencing is not yet a definite tool for bacterial detection, and a clinician's impression on a patient with various evidence, such as clinical history, vital signs, neurologic examination, and CSF profile, should precede clinical decisions.

Nevertheless, the accurate identification of pathogens by superior sensitivity of 16S amplicon sequencing over conventional culture tests could help clinicians manage patients after neurosurgery. Accurate detection of pathogens, even at low bacterial titers, would serve as a guide for antibiotic maintenance and discontinuation. Therefore, nanopore 16S amplicon sequencing is expected to contribute to evidence‐based medicine of neurocritical care in the near future, as additional studies with larger numbers of patients are validated by multiple centers.

## Conflicts of Interest

The authors declare that they have no competing interest.

## Ethical Publication Statement

The authors confirm that they have read the journal's position on issues involved in ethical publication and affirm that this report is consistent with those guidelines.

## Author Contributions

Y.J., S.K., and J.M. wrote and revised the manuscript. Y.J., N.K., and J.M. analyzed the data. Y.J. and S.K. prepared the figures and Tables. J.M., H.S., E.J.H., E.J.K., J.H.P., C‐K.P., J.E.K., and S‐K.K. collected the samples and raw data. S.K.L, W‐S.C., J.M., and K.C. conceptualized and administered the study. W‐S.C., J.M., and K.C. critically reviewed the manuscript.

## Supporting information


**Data S1** This file includes the details of 16S rDNA PCR, nanopore sequencing, cloud‐based data analysis, and determination of genuine infection.

**Table S1** Demonstrates a list of pathogens observed by 16S rDNA sequencing and culture, respectively. The pathogens were grouped by genus and subgrouped by species. The number next to genus and species of bacteria is the number of samples in which the bacteria were isolated. The number in parentheses means the number of false‐positive results.
**Table S2.** Showed false‐positive cases of culture.
**Table S3.** Exhibited the time interval between 16S rDNA sequencing and culture among 17 patients who had positive results both in the two different methods. 16S sequencing was faster than culture study in 16 patients (94.1%). In only one case (5.9%), the culture report was followed by the sequencing report due to the weekend.
