# Supplementary material for: Nanopore 16S sequencing enhances the detection of bacterial meningitis after neurosurgery
Source: Ann Clin Transl Neurol. 2022 Feb 6;9(3):312–25. doi: 10.1002/acn3.51517 (PMC8935320; doi:10.1002/acn3.51517)
Supplement: Supplementary file 1 — Data S1 This file includes the details of 16S rDNA PCR, nanopore sequencing, cloud‐based data analysis, and determination of genuine infection. [file ACN3-9-312-s002.docx]

**Supplementary Materials**

**DNA extraction and 16S rDNA PCR**

DNA was extracted from the CSF specimens and/or the surgical specimens using the PureLink Genomic DNA Mini Kit (Invitrogen, Carlsbad, CA, USA). 16S rDNA polymerase chain reaction (PCR) was performed as described previously^11^. Using the Bacterial 16S rDNA PCR Kit (Takara, Tokyo, Japan), PCR amplified the full-length of 16S rDNA. The genomic DNA was mixed with the 16S rDNA primer mix (Takara, Tokyo, Japan). The DNA was initially denatured at 94°C for 1 min, and then PCR was performed with 35 cycles of 94°C for 30 s, 55°C for 30 s, and 72°C for 1 min with a final extension at 72°C for 3 min in a C1000^TM^ thermal cycler (Bio-Rad, CA, USA). In every PCR, a positive control (bacterial genomic DNA) and a negative control (distilled water) were included. Electrophoresis was performed for the PCR products on a 1.5% agarose gel containing 0.05 μl/ml RedSafe^TM^ (Intron Biotechnology, Seoul, South Korea). The PCR products were visualized using a Bio-Rad Gel Doc EZ Imager. When a PCR-positive band existed in the negative control, contamination was suspected as the cause, and the PCR was repeated from the initial step.

**Nanopore library preparation and sequencing**

When the 16S rDNA PCR of the sample was positive, the preparation of sequencing libraries from the PCR products was performed using the Rapid Barcoding Sequencing Kit (SQK-RBK004, ONT, Oxford, UK). Using the Ultra II End-prep Enzyme (NEB, Hitchin, Hertfordshire, UK), the end-repaired and A-tailed input DNA was incubated at 20°C for 5 min and at 65°C for 5 min. With AMPure XP (Beckman Coulter, High Wycombe, UK), purification of the end-prepped DNA was performed. The elution of the DNA was performed in nuclease-free water, and then, at room temperature, the DNA was ligated with a 1D adapter for 10 min using Blunt/TA Ligase Master Mix (NEB, Hertfordshire, UK). Using the magnetic stand, the 1D adapter DNA was purified with Adapter Binding Buffer (ONT, Oxford, UK). Elution buffer (ONT, Oxford, UK) was used to determine the DNA library. The loading of the presequencing mix onto an R9.5 flow cell (FLO-MIN107) was performed in a mix of running buffer with fuel mix and library loading buffer (ONT, Oxford, UK). Finally, sequencing for 2 or 3 hours and basecalling using MinKNOW software were performed.

**16S amplicon analysis and determination of genuine infection**

Using the cloud-based Metrichor/EPI2ME platform (Metrichor Ltd., Oxford, UK), the analysis of the sequenced reads during or after sequencing was performed. The 16S analysis workflow of EPI2ME was used to perform the BLAST basecalled reads against the NCBI 16S bacterial database. Classification of generated reads was performed to specify the bacterial species based on the % coverage and identity. According to the number of aligned reads, the arrangement of the bacteria list was performed in descending order. From the top of this list, clinicians determined the pathogens. Within a certain genus, species identification was confirmed according to the largest number of aligned reads. Genuine infection was determined when the pathogens identified by the conventional culture study and the sequencing were identical. In cases with inconsistent results between the two methods, genuine infection was clinically determined.
