# Supplementary material for: Nanopore 16S sequencing enhances the detection of bacterial meningitis after neurosurgery
Source: Ann Clin Transl Neurol. 2022 Feb 6;9(3):312–25. doi: 10.1002/acn3.51517 (PMC8935320; doi:10.1002/acn3.51517)
Supplement: Supplementary file 2 — Table S1 Demonstrates a list of pathogens observed by 16S rDNA sequencing and culture, respectively. The pathogens were grouped by genus and subgrouped by species. The number next to genus and species of bacteria is the number of samples in which the bacteria were isolated. The number in parentheses means the number of false‐positive results. Table S2. Showed false‐positive cases of culture. Table S3. Exhibited the time interval between 16S rDNA sequencing and culture among 17 patients who had positive results both in the two different methods. 16S sequencing was faster than culture study in 16 patients (94.1%). In only one case (5.9%), the culture report was followed by the sequencing report due to the weekend. [file ACN3-9-312-s001.docx]

| **Supplementary Table 1. A list of pathogens observed by 16S rDNA sequencing and culture studies** | | | | |
| --- | --- | --- | --- | --- |
| **Isolated bacteria**  **with Nanopore 16S rDNA sequencing (n=40)** | |  | **Isolated bacteria**  **with Culture (n=22)** | |
| **Staphylococcus** | **20** |  | **Staphylococcus** | **11 (2)*** |
| *S. aureus* | 5 |  | *S. aureus* | 3 (1)* |
| *S. caprai* | 5 |  | *S. caprai* | 3 |
| *S. epidermis* | 5 |  | *S. epidermis* | 3 (1)* |
| *S. hominis* | 3 |  | *S. hominis* | 1 |
| *S. capitis* | 2 |  | *S capitis* | 1 |
| **Klebsiella** | **6** |  | **Klebsiella** | **2** |
| *K. pneumoniae* | 3 |  | *K. pneumoniae* | 0 |
| *K. aerogenes* | 3 |  | *K. aerogenes* | 2 |
| **Enterococcus faecium** | **3** |  | **Enterococcus faecium** | **0** |
| **Streptococcus** | **2** |  | **Streptococcus** | **1** |
| *S. pneumoniae* | 1 |  | *S. pneumoniae* | 0 |
| *S. orals* | 1 |  | *S. orals* | 1 |
| **Pseudomonas aeruginosa** | **2** |  | **Pseudomonas aeruginosa** | **2 (1)*** |
| **Serratia nematodiphilia** | **2** |  | **Serratia nematodiphilia** | **2** |
| **Escherichia coli** | **1** |  | **Escherichia coli** | **1** |
| **Moraxella osloensis** | **1** |  | **Moraxella osloensis** | **0** |
| **Corynebacterium** | **1** |  | **Corynebacterium** | **0** |
| **Candida albicans** | **1** |  | **Candida albicans** | **1** |
| **Acinetobacter** | **1** |  | **Acinetobacter** | **1** |
|  |  |  | **Unidentified Gram (+) rod** | **1 (1)*** |
| *The number in parentheses means the number of false-positive results. | | | | |

| **Supplementary Table 2. False-positive cases of culture study** | | |
| --- | --- | --- |
| **Case** | **Culture** | **Sequencing** |
| **1** | *Pseudomonas aeruginosa* | None |
| **2** | *Staphylococcus aureus* | None |
| **3** | *Unidentified Gram (+) rod, non-spore-forming* | None |
| **4** | *Staphylococcus epidermidis* | None |

| **Supplementary Table 3. Time interval between 16S rDNA sequencing and culture studies**. | | | | | | | |
| --- | --- | --- | --- | --- | --- | --- | --- |
| **Sex** | **Age** | **Culture date** | **Sequencing date** | **Culture report time** | **Sequencing report time** | **Time interval (hours)*** | **Holiday or Weekend†** |
| F | 69 | 2019-03-18 | 2019-03-21 | 2019-03-26 15:06 | 2019-03-18 17:00 | 190.1 | No |
| M | 21 | 2019-11-18 | 2019-11-20 | 2019-11-27 14:20 | 2019-11-20 19:00 | 163.3 | No |
| M | 63 | 2020-06-09 | 2020-06-10 | 2020-06-16 17:25 | 2020-06-11 10:00 | 127.4 | No |
| F | 52 | 2019-05-29 | 2019-05-29 | 2019-06-04 13:49 | 2019-05-30 9:00 | 124.8 | No |
| F | 56 | 2018-12-27 | 2018-12-28 | 2019-01-01 16:04 | 2018-12-28 13:00 | 99.1 | No |
| F | 76 | 2018-08-13 | 2018-08-14 | 2018-08-18 13:50 | 2018-08-14 13:00 | 96.8 | No |
| M | 73 | 2018-12-06 | 2018-12-07 | 2018-12-11 13:46 | 2018-12-07 16:50 | 92.9 | No |
| F | 64 | 2019-11-20 | 2019-11-21 | 2019-11-25 14:34 | 2019-11-22 10:30 | 76.1 | No |
| M | 64 | 2018-01-15 | 2018-01-15 | 2018-01-18 17:19 | 2018-01-15 17:40 | 71.7 | No |
| M | 63 | 2020-06-04 | 2020-06-08 | 2020-06-11 18:30 | 2020-06-08 19:00 | 71.5 | Yes |
| M | 37 | 2018-05-23 | 2018-05-25 | 2018-05-28 17:20 | 2018-05-25 19:00 | 70.3 | No |
| M | 6 | 2020-05-12 | 2020-05-13 | 2020-05-16 16:08 | 2020-05-13 19:00 | 69.1 | No |
| M | 2 | 2018-11-28 | 2018-12-03 | 2018-12-06 10:24 | 2018-12-03 19:00 | 63.4 | Yes |
| M | 61 | 2019-04-22 | 2019-04-25 | 2019-04-27 13:22 | 2019-04-25 19:00 | 42.4 | No |
| M | 28 | 2018-02-18 | 2018-02-20 | 2018-02-21 11:19 | 2018-02-20 14:00 | 21.3 | No |
| M | 75 | 2018-11-26 | 2018-11-27 | 2018-11-28 10:33 | 2018-11-27 19:00 | 15.6 | No |
| F | 66 | 2018-08-16 | 2018-08-20 | 2018-08-18 13:49 | 2018-08-20 19:00 | -53.2 | Yes |
| *Time interval = (Culture report time – Sequencing report time); the positive value means that the sequencing report preceded the culture report.  † ‘Holliday or Weekend’ means that the sequencing result report was delayed due to holiday or weekend when the 16S rRNA gene sequencing was unavailable. | | | | | | | |
